# The Contribution of Endogenous Modulatory Systems to TMS- and tDCS-Induced Analgesia: Evidence from PET Studies

**DOI:** 10.1155/2018/2368386

**Published:** 2018-11-13

**Authors:** Marcos F. DosSantos, Aleli T. Oliveira, Natália R. Ferreira, Antônio C. P. Carvalho, Paulo Henrique Rosado de Castro

**Affiliations:** ^1^Instituto de Ciências Biomédicas (ICB), Universidade Federal do Rio de Janeiro (UFRJ), Rio de Janeiro, Brazil; ^2^Programa de Pós-Graduação em Radiologia, Universidade Federal do Rio de Janeiro (UFRJ), Rio de Janeiro, Brazil; ^3^D'Or Institute for Research and Education, Rio de Janeiro, RJ, Brazil

## Abstract

Chronic pain is an important public health issue. Moreover, its adequate management is still considered a major clinical problem, mainly due to its incredible complexity and still poorly understood pathophysiology. Recent scientific evidence coming from neuroimaging research, particularly functional magnetic resonance (fMRI) and positron emission tomography (PET) studies, indicates that chronic pain is associated with structural and functional changes in several brain structures that integrate antinociceptive pathways and endogenous modulatory systems. Furthermore, the last two decades have witnessed a huge increase in the number of studies evaluating the clinical effects of noninvasive neuromodulatory methods, especially transcranial magnetic stimulation (TMS) and transcranial direct current stimulation (tDCS), which have been proved to effectively modulate the cortical excitability, resulting in satisfactory analgesic effects with minimal adverse events. Nevertheless, the precise neuromechanisms whereby such methods provide pain control are still largely unexplored. Recent studies have brought valuable information regarding the recruitment of different modulatory systems and related neurotransmitters, including glutamate, dopamine, and endogenous opioids. However, the specific neurocircuits involved in the analgesia produced by those therapies have not been fully elucidated. This review focuses on the current literature correlating the clinical effects of noninvasive methods of brain stimulation to the changes in the activity of endogenous modulatory systems.

## 1. Introduction

According to the International Association for the Study of PAIN (IASP), pain is “an unpleasant sensory and emotional experience associated with actual or potential tissue damage, or described in terms of such damage” [1]. Based on the temporal progress, it can also be classified in acute and chronic. While acute pain is clearly related to tissue injuries, chronic pain persists even after the resolution of a primary lesion or a tissue damage and turns into a self-maintained disease [2], with great impact on the patient's quality of life and huge economic effects. For example, only in the US, chronic pain affects approximately 100 million people at a cost of $635 billion annually [3]. Nevertheless, the mechanisms underpinning the transition from acute to chronic pain are still poorly comprehended. With the enhancement of neuroimaging methods, it has been possible to better understand brain structures related to the central processing of acute and chronic pain as well as the specific regions associated with the endogenous modulation of painful stimuli [4]. Such structures form a sophisticated neural network responsible for the so-called sensitive-discriminative, affective-motivational, and cognitive-evaluative dimensions of the pain experience [5].

Due to the heterogeneous etiologies, complicated pathophysiological mechanisms and challenging clinical management of chronic pain syndromes, modern technologies have been developed to enhance the efficacy of the therapeutic strategies [6]. Among those novel therapeutic approaches, noninvasive neuromodulatory techniques have emerged as promising clinical alternatives.

Noninvasive methods of brain stimulation such as transcranial electrical stimulation (tDCS) and transcranial magnetic stimulation (TMS) have been largely applied to induce long-lasting changes in the neural activity. Considering the abilities of such methods to modulate the cortical excitability without producing substantial side effects and their potential roles in promoting neuroplasticity, their clinical effects have been extensively investigated in the last few years [7–10]. Hence, several therapeutic applications have been proposed, including poststroke motor rehabilitation, depression treatment, cognitive training, and pain management [11–15].

In fact, electrical brain stimulation has a long history. The first reports date back to ancient Greece, when the electric fish was used to treat headaches and the eleventh century when it was used to treat epilepsy [16]. Several years later, after the discovery that weak electrical stimulation can modulate the cortical excitability [17], experimental and clinical studies started to investigate its potential psychotherapeutic properties [18–20]. Nonetheless, only after the advent of the electric batteries in the eighteenth century, those methods have become widespread in the treatment of neurological diseases and psychiatric disorders, a clinical application that has become routinely used in the course of the nineteenth century and especially during the beginning of the twentieth century [21]. On the contrary, the concept of TMS was only introduced in the 1980s [22]. During the following years, the effects of tDCS on the cortical excitability modulation could be demonstrated [23, 24]. Thereafter, many clinical applications for TMS and tDCS have emerged, and one of those include pain treatment, especially in cases of chronic refractory pain [25].

The central modulatory mechanisms involved in pain perception and modulation are complex. They comprise multiple neurotransmitters and neural pathways. However, recent studies have unveiled that the endogenous opioid system is probably one of the chief modulatory mechanisms related to this process [26–30]. Based on this concept, some studies started to investigate the possible correlation between the analgesic effects driven by noninvasive electrical therapies of neuromodulation (tDCS and TMS) and the activity of endogenous pain modulatory systems [26–32].

## 2. Changes in the Endogenous Modulatory Systems Functioning Related to Chronic Pain

Pain processing is complex and involves an extensive network of brain structures referred in the past as pain neuromatrix [33]. The results of functional neuroimaging studies allowed the separation of those structures into a medial and a lateral pain pathway. The lateral pathway comprises the primary somatosensory cortex (SI), the posterior insula, and the parietal operculum. On the contrary, the medial pathway includes the anterior insula, thalamus, anterior cingulate cortex (ACC), and prefrontal cortex (PFC). The lateral pain pathway would process the sensory features of the pain experience whereas the medial pathway would be involved in the processing of the emotional aspects of pain [34–36].

Nonetheless, this can be considered very simplistic, since the concepts of nociception and pain have been entirely revised and modified during the last decades. Therefore, the definition of pain as a unidirectional process, restricted to nociception as well as the anatomical concept of a “pain neuromatrix” has been progressively replaced throughout the years. In addition, recent scientific evidence indicate that those pain-related brain regions, which were previously included in the “pain neuromatrix,” are, in fact, not exclusively engaged in the processing of nociceptive information but also in the detection of other sensory stimuli [37]. In this regard, functional resonance imaging (fMRI) and electroencephalography (EEG) studies compared brain responses related to different modalities of sensory stimuli (e.g., tactile, auditory, and visual stimuli). The results showed that the responses produced by nociceptive stimuli represent multimodal neural activity in the ACC, parietal operculum, posterior parietal cortex, and the insula. On the contrary, brain areas that specifically responded to nociceptive stimuli were sparsely found in the brain [5, 38, 39]. Moreover, there is a vast literature showing that pain-related brain regions actively contribute the endogenous pain modulation [40]. Therefore, differences in the functioning of such structures help to explain the individual variations in the endogenous pain control, a key element to the development of chronic pain.

The nociceptive input, in its course from the peripheral to the central nervous system (PNS and CNS, respectively) is constantly regulated by highly specialized modulatory systems. Several aspects of this endogenous control of the nociceptive information have been recognized for over 100 years [41, 42]. However, only in the recent years have its mechanisms been deeply investigated, with especial attention to the anatomical components of the brainstem reticular formation and the descending pain inhibitory system.

**Figure 1:**
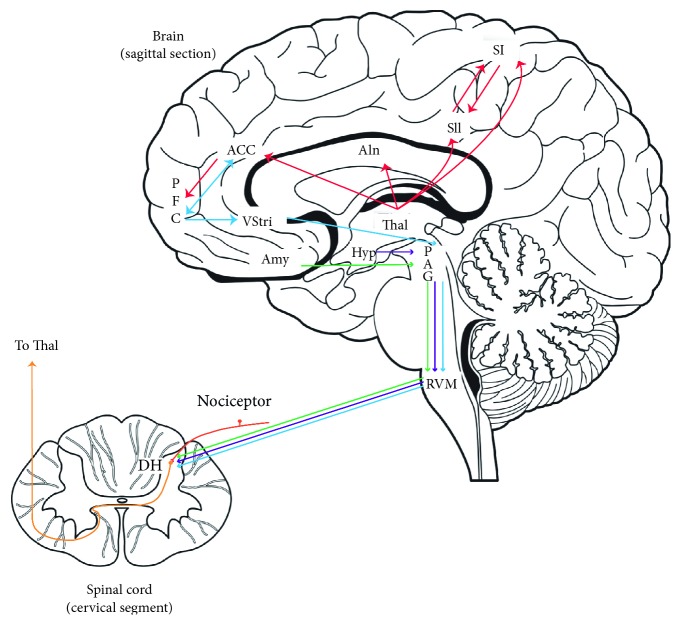
An overview of the major components and connections of the pain modulatory system. Nociceptive inputs ascending from dorsalhorn (DH) of the spinal cord to the ventroposterolateral or ventroposteromedial nuclei of thalamus, from where it flows in different pathways: (1) lateral thalamus to SI and SII—processing the sensory-discriminative aspect of pain; (2) medial thalamus to AIn, ACC, and PFC (viaACC)—processing the affective-motivational component of pain. The descending pain modulatory regulation involves the components of the reticular formation (PAG and RVM) which can modulate nociceptive signals at the DH of the spinal cord. This process is highly regulated by the opioidergic and serotonergic systems. RVM: rostroventromedial medulla; PAG: periaqueductal gray; Thal: thalamus; HT: hypothalamus; Amy: amygdala; VStri: ventral striatum; AIn: anterior insula; ACC: anterior cingulate cortex; PFC: prefrontal cortex; SI:primary somatosensory cortex; SII: secondary somatosensory cortex; DH: dorsal horn of the spinal cord. Based on Morton et al. [36], Fields [46], and Jones and Brown [64].

The descending pain modulation is performed through a broad network of cortical areas, with the fundamental recruitment of subcortical structures, including the components of the reticular formation, such as the periaqueductal gray matter (PAG), rostroventromedial medulla (RVM), nucleus raphe magnus, and locus coeruleus. Thalamus and hypothalamus also participate in this process. Most of those structures can play important roles not only in pain modulation but also in pain processing. For instance, ventrolateral PAG acts as an integrative structure for neural inputs arising from the cerebral cortex, spinal cord, and brainstem itself [43]. Furthermore, it modulates nociceptive signals, acting directly at the dorsal horn of the spinal cord [44] or indirectly, through connections with the dorsolateral pontine tegmentum (DLPT) and RVM [45], which in turn projects to the dorsal horn neurons that process the nociceptive information [46]. An overview of pain processing and modulation is illustrated in the Figure 1. Nonetheless, this mechanism can be considerably more complex, involving a spinal-supraspinal-spinal loop [47]. Moreover, it has been shown that the PAG activity, with the important regulation of the cingulate cortex, is highly correlated to the attention directed toward painful stimuli [48, 49]. The contribution of several neurochemical mediators such as serotonin (5-hydroxytryptamin or 5-HT), endogenous opioids, dopamine, glutamate, and gamma-aminobutyric acid (GABA) to the endogenous pain modulation has been also vastly reported [49, 50].

### 2.1. Opioid System

Due to its effects in pain modulation, the opioid system has been a frequent target of both experimental and clinical pain studies. In fact, a large concentration of all opioid receptors subtypes, *µ* (MOR), *δ* (DOR), *κ* (KOR), and ORL1, is found in an extensive network of brain structures such as the ACC, midcingulate cortex (MCC), insula, PFC, basal ganglia, amygdala, hypothalamus, DLPT, PAG, RVM, and spinal cord. Those receptors are activated by endogenous opioids as well as synthetic opiates, resulting in analgesic effects [51–53]. The functioning of those structures and the interdependence between them have proven to be crucial for the analgesic effects produced by opioids. For example, it has been described that MOR-induced analgesia depends on the integrity of the RVM neurons that project to the spinal cord [46]. In addition, there is also evidence that activation of the RVM induced by opioids can be predicted by the rostral ACC (rACC)-PAG coupling [52].

Despite the indisputable evidence regarding the contribution of the opioid system to pain modulation, some studies have also suggested that the activity of opioid receptors is more associated with the learning prediction over time rather than the painful outcome [54]. Moreover, the role of the opioid system in inhibition of fear acquisition has been previously reported by one study that showed the administration of the MOR antagonist naloxone in healthy subjects increases fear acquisition and changes activation profile in the amygdala [52]. It has also been demonstrated that pain expectations may directly affect the nociceptive processing [55, 56]. Such findings contribute to the placebo effect, which has been proved to be opioid-mediated [53]. For instance, it has been shown that naloxone administration reduces neural and behavioral placebo effects as well as placebo-induced responses in several cortical and subcortical areas that constitute the descending pain modulatory system (e.g., rACC, PAG, RVM, and hypothalamus). In addition, it abolished the rACC-PAG coupling induced by the placebo intervention [52]. The expectation of placebo-induced analgesia positively correlates to MOR availability [53]. On the contrary, negative suggestion reduces the analgesic effects of synthetic opiates [57]. All those findings support the close association between opioids and expectations that drive placebo-mediated analgesia [55].

Overall, the top-down pathways involved in the opioid-mediated antinociception have been recently summarized as follows: (1) PFC and ACC interact to limbic areas to provide contextual cognitive information. Indeed, opioid signaling in the rACC plays a crucial role in the relief of pain aversiveness [58]; (2) amygdala and insula are important for aversive learning; (3) the ventral striatum plays a critical role in rewarding learning. Bidirectional modulation of the ventral striatum is important to reduce or increase nociception, which depends on opioidergic connections from the ACC. Remarkably, it has also been reported that the connectivity between ventral striatum and PFC predicts the prognosis of chronic pain [59], and (4) all cortical inputs converge to the PAG-RVM-spinal cord, which facilitates or inhibits nociception [46, 54]. However, there are still many questions that must be addressed regarding the mechanisms of opioid-induced analgesia, especially in patients with chronic pain syndromes, which have started to be exposed in the last 20 years.

The advent of positron emission tomography (PET) and more specifically, the improvement in the synthesis of selective radioligands has allowed researchers to investigate the physiological functioning of the endogenous modulatory system, especially the dopaminergic and opioidergic systems, *in vivo*, and more important, to correlate the functioning of those systems to experimental acute pain and also to chronic pain syndromes.

In one of the pioneer studies using selective (MOR) radioligands, a decreased nondisplacable binding potential (*BP*_ND_) of the MOR selective radiotracer [11C] carfentanil was found in several brain areas directly related to different aspects of pain, including the nucleus accumbens (NAc), amygdala, and dorsal ACC (dACC), of patients diagnosed with fibromyalgia syndrome, an idiopathic chronic pain disorder of obscure etiology and difficult control. Such remarkable outcomes might indicate a higher release of endogenous opioids related to the modulation of the nociceptive information or alternatively, a downregulation of MOR due to a prolonged pain experience [60]. In the same work, the MOR *BP*_ND_ within the NAc was negatively correlated to with the ratings of the affective dimension assessed through the short form of the McGill pain questionnaire (SF-MPQ) [61]. Those findings were in line with previous evidence that indicated a decreased *BP*_ND_ of opioid receptors, evaluated through the nonselective radiotracer [11C] diprenorphine, in other chronic pain disorders [62, 63], with regularization driven by pain decrease [62, 63]. They also support the hypothesis that the activation of the opioid system in chronic pain condition takes place mainly in the medial pain system [54].

On the contrary, other studies found increased availability of opioid receptors related to chronic pain. In one of these studies, the higher availability of opioid receptors was detected in the caudate nucleus, NAc and subcallosal area, insula, and PAG in chronic pain due to osteoarthritis and rheumatoid arthritis when compared to healthy subjects. The mechanism involved in this process could be greater density of opioid receptors on neuronal cellular membranes, reduced endogenous opioid release, or even both. However, the same study showed a positive association between the availability opioid receptors in the caudate nucleus and the acute thermal pain thresholds in both patients and healthy controls. The authors of that study concluded that an adaptive upregulation of opioid receptors takes place in response to chronic pain. Therefore, increased BP of opioid receptors would be associated with higher pain threshold and greater pain resilience. The contradictory results regarding the availability of opioid receptors in chronic pain among different PET studies could be related to the selectivity of the radioligands to the different subtypes of opioid receptors, mu, kappa, or delta [64].

Studies that combine PET and fMRI have provided insights into the mechanisms that determine the role of endogenous opioids in the modulation of nociceptive inputs and also how altered functioning of the opioidergic system plays a role in chronic pain pathophysiology. In one of these studies, colocalized changes in the BOLD (blood oxygen level dependent) signal, measured through fMRI and changes in the availability of opioid receptors, evaluated through the *BP*_ND_ of [11C] diprenorphine, have been found in the thalamus of healthy subjects undergoing pressure painful stimuli. These findings suggest that thalamic endogenous opioid release induced by noxious stimuli (in this case, pressure pain) produces thalamic inhibition of thalamic neurons and ultimately contributes to the changes observed in the BOLD fMRI signals [65]. Such information has been applied to a chronic pain disorder by a recent study that has demonstrated a significant positive association between MOR availability, measured by PET and brain activity evoked by pain, evaluated through fMRI in the dorsolateral prefrontal cortex (DLPFC) and rostral anterior cingulate cortex (rACC) of fibromyalgia patients. In addition, a negative association between MOR *BP*_ND_ and BOLD signals evoked by pain and the affective/sensory pain ratio was found in the medial frontal gyrus (MFG) and in different parts of the cingulate cortex [66]. Based on such results, it has been proposed that, in fibromyalgia patients, tonic increased levels of endogenous opioids would lead to downregulation or lower affinity of MOR on GABAergic interneurons located within brain areas that participate in pain control, including the PFC and the ACC. Therefore, the inhibition of GABAergic interneurons triggered by phasic endogenous opioid release induced by noxious stimuli, an important physiological mechanism of antinociceptive neuron disinhibition and consequent descending modulatory system activation, could possibly be compromised in fibromyalgia patients. It has also been hypothesized that, in healthy subjects, the same levels of MOR *BP*_ND_ would result in higher BOLD signals in pain-related structures, when compared to fibromyalgia patients. However, the presence of this mechanism has not been investigated in healthy subjects, which limits a broader interpretation of those results.

Recent PET studies have also demonstrated altered functioning of MOR and mu-opioid neurotransmission in trigeminal neuropathic pain and migraine headaches. In trigeminal neuropathic pain, a reduced availability of MOR was detected in the ventral striatum, specifically in the NAc [27], a key component of the reward system, with high concentrations of opioid receptors [67]. On the contrary, in migraine headaches, a reduced MOR *BP*_ND_, representing activation of the mu-opioid neurotransmission occurred in the medial prefrontal cortex (mPFC) during headache episodes (itcal phase) when compared to the headache-free periods (interictal phase) [68]. In effect, PFC activation had been also demonstrated in both induced and spontaneous migraine headache attacks [69, 70]. In another PET study, the activation of MOR in the PAG and red nucleus of migraine patients were positively correlated to the occurrence of thermal allodynia induced by heat stimulation in the territory of the ophthalmic (V1) division of the trigeminal nerve [71].

Taken together, the results of those studies suggest a competition of opioid receptors driven by chronic pain [36]. If true, this concept would support a potential therapeutic use of enkephalinase inhibitors (DENKIs), which have been developed to increase the half-lives and extracellular concentrations of enkephalins, in the treatment of chronic pain [72].

**Figure 2:**
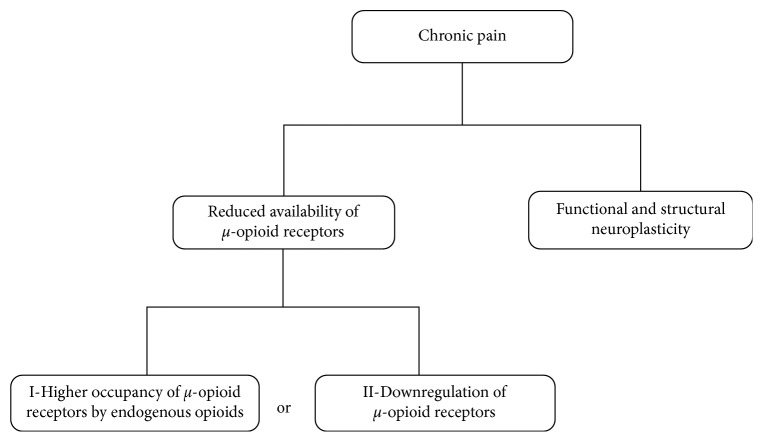
Possible mechanisms underlying changes in the opioid system induced by chronic pain.

The decreased *BP*_ND_ associated with acute or chronic pain could indicate an increased release of endogenous opioids needed to the descending modulation of the nociceptive information or alternatively, a downregulation of MOR due to a sustained and constant pain experience [60]. These hypotheses are illustrated in Figure 2. This intriguing question has been recently investigated in an experimental model of peripheral neuropathy. The results confirmed the decreased opioid receptor availability, using a nonselective opioid radiotracer, within the caudate-putamen and motor cortex of the animals that underwent peripheral nerve injury, when compared to controls that underwent sham surgery. A further immunohistochemistry analysis showed lower expression of MOR in the basal ganglia and insula of neuropathic pain animals. However, such an altered expression of MOR was not followed by changes in the opioid peptide enkephalin or in the neuronal marker NeuN. Such results indicate that the decreased opioid receptor availability found in the animal model of neuropathic pain is driven by changes in the expression of MOR rather than variations in levels of enkephalins or in the density of opioidergic neurons [73]. In addition, they strongly suggest that the PET findings reported by clinical studies are related to the pain itself and not to other factors (genetics or treatment) [74].

### 2.2. Dopaminergic System

Dopamine is a catecholamine neurotransmitter present in both in the CNS and in the PNS. In the CNS, dopamine participates in diverse functions such as motor control, reward system as well as pain transmission and modulation [75, 76]. Dopamine acts at D1 receptors (DRD1), leading to cAMP formation and protein kinase activation, with consequent pronociceptive effects. On the contrary, activation of dopamine receptor D2 (DRD2) has opposite effects, thus producing antinociceptive effects [77]. The roles of the ventral and dorsal basal ganglia dopaminergic neurotransmissions in the pain experience have been explored. In this regard, a previous study found a positive correlation between the activation of the nigrostriatal neurotransmission (caudate nucleus and putamen) mediated by DRD2 and the sensory/affective dimensions of pain, while the activation of the mesolimbic (nucleus accumbens) dopamine pathway (DRD2 and DRD3) was only correlated to the emotional pain dimension [78]. Another study showed that the dopaminergic system encodes and regulates motivational valence and salience of painful stimuli, therefore contributing to the decision of whether the painful stimulus should be endured or not [79].

The existence of dynamic fluctuations in the activity of the dopaminergic system of migraine patients has also been demonstrated with PET [80]. Notwithstanding the striatal *BP*_ND_ of the dopamine ligand [11C] raclopride, a selective DRD2/DRD3 radiotracer did not differentiate migraine patients and healthy subjects at a baseline condition, a significant increase of [11C] raclopride *BP*_ND_ in the striatum (ipsilateral caudate and bilateral putamen), which putatively represents a decrease in dopamine release, was found during the itcal phase and thermal allodynia, when compared to their corresponding interictal phases. Such results were interpreted as a provisional shift of dopamine receptors occupancy instead of a variation in the number of receptors available [80]. Moreover, a rapid reduction of [11C] raclopride *BP*_*ND*,_ representing a sudden increase in the release of endogenous dopamine, was elicited by cutaneous thermal allodynia, when compared to the migraine attack phase at rest. Those findings are in agreement with the structural (larger gray matter volume) and functional (lower activation) changes previously detected in the striatum of migraine patients [81].

Changes in the dopaminergic neurotransmission have also been reported in other chronic pain disorders. Nonetheless, contradictory results have been reported. For instance, when compared to healthy controls, fibromyalgia patients exhibited lower availability of dopamine receptors DRs in a previous study [82]. In another work, a reduction of presynaptic dopaminergic activity was demonstrated in several brain regions of fibromyalgia patients [75]. Conversely, in other studies, patients with atypical facial pain and burning mouth syndrome, two chronic neuropathic orofacial pain conditions, displayed higher baseline dopamine receptors availability [83, 84]. A recent study has also found differences in the plasma levels of dopamine in patients diagnosed with myofascial temporomandibular disorders when compared to healthy subjects, suggesting the function of dopamine in the modulation of nociceptive orofacial pain [85]. Such data suggest that an altered functioning of the dopaminergic system plays a relevant role in the mechanism of chronic pain. However, the direction of those changes cannot be generalized since it seems to be highly specific for each group of chronic pain syndromes (e.g., nociceptive or neuropathic) and even in each particular disease. Supporting this hypothesis, the evaluation of the dopaminergic function in a cohort of chronic back pain patients (CBP) revealed a baseline reduction in the *BP*_ND_ of DRD2/DRD3 within the ventral striatum, when compared to controls, and the same change reported in fibromyalgia patients. Furthermore, in the CBBP study, this decrease in the *BP*_ND_ of DRD2/DRD3 was negatively correlated to pain sensitivity and positively correlated to pain tolerance and to the positive affect of pain [81]. Those findings also corroborate the association found between the activation of DRD2/DRD3 in the dorsal striatum and the ratings of the sensory/affective aspects of experimental pain, in healthy volunteers [78]. Interestingly, a coupling between DRD2/DRD3 *BP*_ND_ and MOR *BP*_ND_ was detected in the amygdala related to experimental pain, which indicates a potential interaction between the dopaminergic and opioidergic systems in the ventral striatum, contributing to the development of chronic pain.

Facilitation of NAc dopamine release has also been linked to the activity of VTA or NAc neurons [86]. In addition, an interaction between opioidergic and dopaminergic systems has been shown in the dorsal hippocampus, a region rich in opioid neurons and receptors [87] and that receives dopaminergic projections from the substantia nigra and from the ventral tegmental area (VTA), in an experimental model formalin-induced orofacial pain. Interestingly, opioid blockade with naloxone reversed dopamine-induced antinociceptive effects. Nonetheless, neither D1 nor D2-like receptors antagonists promoted significant effects on the antinociception produced by morphine [88]. A possible role of this cross-talk between opioidergic and dopaminergic systems, in the opioid tolerance and dependence, has also been suggested [81]. However, this hypothesis must be further explored.

The growing evidence supporting the presence of neuroplastic changes affecting major endogenous modulatory mechanisms (e.g., opioidergic and dopaminergic) in different chronic pain disorders raises the possibility that neuromodulatory therapies can act by modifying the activity and possibly reverting the maladaptive neuroplasticity that alters the physiologic functioning of those systems.

## 3. An Overview of Noninvasive Neuromodulatory Techniques

TMS technique modulates the cortical activity through the generation of a magnetic field that passes through a coil. The induced current runs parallel to the plane of the coil and reaches the scalp and skull, producing action potentials that will excite or inhibit the activities of cortical and potentially subcortical areas and related neural network [89, 90]. The frequency varies from low (≤1 Hz) to high (>5 Hz). Overall, high frequencies produce excitatory effects, and it has been hypothesized that it increases the synapse efficiency by inducing long-term potentiation (LTP) [91]. On the contrary, low frequencies produce inhibitory effects, putatively through long-term depression (LTD) [92]. Therefore, LTP and LTD, well-known mechanisms of synaptic plasticity would constitute the neuroanatomical basis of TMS mechanisms. Either high or low frequencies have been proved to promote analgesic effects. However, this effectiveness highly depends on the target selection, which can be explained by the great complexity of the pain-related neural networks [89]. In this respect, the M1 has been elected as the main cortical target for pain treatment [26, 30, 93, 94]. Nevertheless, other regions have also been adopted as TMS targets. Those areas include the occipital cortex, the dorsolateral prefrontal cortex (DLFPC), and the dorsal anterior cingulate cortex (dACC) [95–97]. Notwithstanding the processes involved in the analgesic effects of TMS are still not established, both cortical and subcortical effects have been suggested [98, 99]. In addition, the results of some studies suggest that the endogenous opioid system might play a prominent role [26, 89, 94, 100].

TDCS is another neuromodulatory technique capable of modifying the action potentials of neurons, thus producing changes in the excitability of neural circuits [101]. This method comprises the direct application of low-intensity electric current to the scalp that flows between two electrodes, an anode (positive pole) that has an excitatory cortical effect that results in neuronal depolarization and a cathode (negative pole) that decreases the cortical excitability and induces hyperpolarization [24]. The duration and direction of these effects depend on several parameters such as the electrode size, polarization, and more important, the position of the electrodes applied on the scalp, that will in turn, determine the direction and the spatial distribution of the resultant electric current. Other important properties that must be considered are the intensity of the electric current and the duration of the stimulus [101, 102]. Likewise TMS, M1 is the most frequent tDCS target in the majority of pain studies. It has been suggested that the analgesic effects of tDCS involve changes in the corticospinal excitability of brain regions related to pain modulation, with also a critical participation of endogenous pain modulatory systems [28, 94]. An overview of the current literature and the main studies investigating the participation of endogenous modulatory systems in the TMS-tDCS-induced analgesia is found in Tables 1–4.

## 4. Effects of tDCS and TMS on Endogenous Pain Modulatory Systems

The endogenous opioid system is one of the key components of the antinociceptive physiological mechanisms [103–105]. Hence, its participation in the analgesic effects of noninvasive methods of transcranial electrical stimulation has been explored in the last few years. The primary evidence regarding the role of the opioid system in the analgesia obtained with neurostimulation procedures comes from an experimental research model. In that study, the analgesic effects of tDCS were prevented by the administration of naloxone, a potent opioid antagonist [106]. The outcomes of this initial proof-of-concept study correlates to those of a clinical research carried out a decade later that found increased plasmatic levels of beta-endorphin in patients undergoing active tDCS, but not in a placebo group [107].

The improvement of neuroimaging methods during the last decades also allowed the identification of the CNS components and related modulatory systems that contribute to the painful experience and the activation of such structures driven by different neuromodulatory methods. The first clinical study in this matter demonstrated an increased release of endogenous opioids in several brain areas related to pain subsequently to motor cortex stimulation with implanted electrodes (MCS) [108]. Nonetheless, the first study that demonstrated the involvement of the opioidergic system with noninvasive brain stimulation was conducted by de Andrade et al. [26]. In that randomized, double-blind clinical protocol performed in healthy volunteers, the authors compared the effects of TMS given to M1 and DLPFC/PMC with and without naloxone. Their results revealed a significant reduction in the analgesic effects of M1-TMS after the administration of naloxone, suggesting that the activation of descending modulatory pathways within brain areas containing high concentration of MOR such as PAG and RVM might play a role in the analgesic mechanisms of TMS. Interestingly, in the same study, the stimulation of DLPFC/PMC was not altered by the administration of naloxone, indicating that distinct mechanisms could be associated with M1 and DLPFC TMS and that the activation of the endogenous opioid system would not play a significant role in the analgesia promoted by DLPFC-TMS. However, a further study by Taylor et al. found a decrease in the analgesic effects of DLPFC-TMS, when administered combined with naloxone [97]. Those contradictory findings may be explained by the methodological differences between both studies, including the side of stimulation (left DLPFC in Taylor's study and right in de Andrade's study) as well as the doses of TMS and naloxone used (Taylor's study: bolus of 0.1 mg/kg naloxone; de Andrade's study: bolus of 0.1 mg/kg followed by a continuous infusion of 0.1 mg/kg/h until the end of the stimulation).

The aforementioned studies bring indirect evidence regarding the contribution of the endogenous opioid system to TMS effects. With the purpose to expand this concept and to provide direct evidence of this possible relationship, some studies have used PET in order to elucidate the pathways responsible for the pain modulation related to TMS [30]. The first studies in this subject reported increased dopamine release induced by M1-TMS and PFC-TMS in the striatum [109, 110] as well as in the ACC and orbitofrontal cortex [111]. In addition, the evaluation of the MOR contribution to the TMS-driven analgesia has been explored through PET.

In a randomized, double-blind, sham-controlled, crossover protocol, Lamusuo et al. [30] investigated the effects of TMS on both the opioidergic and the dopaminergic systems, using the specific radiotracers [11C] carfentanil (MOR) and [11C] raclopride (DRD2/DRD3), respectively. The results indicated a significant *BP*_ND_ of [11C] carfentanil in the ACC, mPFC, medial orbitofrontal cortex (mOFC), and ventral caudate and putamen of both cerebral hemispheres, after the application of active TMS, compared to sham. Interestingly, similar results did not occur with the raclopride *BP*_ND_, supporting the hypothesis that the activation of the endogenous opioid neurotransmission and consequent release of endogenous opioids should be one of the most important pathways related to the analgesic effects of M1-TMS [30, 94]. However, it has also raised questions regarding the recruitment of the dopaminergic system by TMS. Although the absence of changes in the [11C] raclopride *BP*_ND_ in the Lamusuo's study [30] suggests that TMS does not promote a significant tonic long-lasting dopamine release, the ipsilateral potentiation of the blink reflex habituation [112], a phenomenon controlled by the nigrostriatal dopaminergic system, produced by TMS, indicates that some dopamine release might have occurred. Moreover, the control of expectation, which was adopted in the Lamusuo's study to avoid a possible interference of expectation on dopamine release [113], is another element that must considered when interpreting those results, since it might have influenced the release of dopamine stimulated by TMS.

Genetic variability in dopamine receptors might also be considered when evaluating the effects of TMS or tDCS on the dopaminergic system. In a previous study, the analgesic effects produced by rTMS applied to S1 were demonstrated in subjects that presented the DRD2 957TT genotype [114]. The authors of that study hypothesized that such effect might be driven by a high-amplitude phasic striatal dopamine release, which in turn would be related to a low tonic dopamine release in 957TT homozygotes [115–117]. According to this concept, dopamine DRD2 polymorphisms could control phasic dopamine release in the striatum and downstream the pathway opioid-induced analgesia.

The contribution of *N*-methyl-d-aspartate (NMDA) glutamate receptors to TMS analgesia has also been studied. Confirming the initial hypothesis of the involvement of the glutamatergic system in TMS-induced analgesia, the injection ketamine, a noncompetitive NMDA antagonist reduced the analgesic effects of rTMS in healthy subjects. Strikingly, this effect was found with both DLPFC and M1 stimulation, suggesting a common pathway for both types of stimulation and involving NMDA receptors. Such results also suggest that a long-term potentiation (LTP)-like phenomenon may underlie TMS effects [118].

With respect to activation of endogenous modulatory mechanisms related to tDCS, only a few PET studies have been performed until this moment, with similar results to those reported with TMS. An initial PET investigation demonstrated a release of endogenous opioids, measured by a decrease in the MOR *BP*_ND_ within the PAG, PFC, anterior thalamus, ACC, and insula after a single session of active M1-tDCS in a postherpetic neuralgia patient [27]. Using a similar methodology, a further work demonstrated mu-opioid activation driven by tDCS. More important, the activation induced by sham and active tDCS occurred in shared (precuneus and PAG) and specific areas (thalamus for sham stimulation and PFC for active tDCS). The activation of PAG and thalamus adds evidence to the involvement of the descending modulatory system in the tDCS-related analgesia [28].

A recent experimental study conducted in an animal model of neuropathic pain showed that the antiallodynic effects of tDCS were not only associated with the opioidergic system but also with the adenosinergic, serotonergic, noradrenergic, cannabinoid, GABAergic, and glutamatergic systems [119]. Interestingly, that was the first study showing the involvement of both cannabinoid receptors (CB1 and CB2) in tDCS-induced analgesia. This might be of particular interest for neuropathic pain treatment, cannabinoid receptors has been associated with neuropathic pain [120, 121]. It has also been demonstrated that anodal tDCS applied to the motor cortex can produce significant analgesic effects in the presence of temporal summation-induced plasticity affecting pain pathways, reinforcing the hypothesis that tDCS can act through the modulation of endogenous top-down mechanisms of pain control [122].

Moreover, a recent article reviewed to which extent, the use of concomitant medication impacts tDCS effects. The results indicate that several classes of medications, including sodium and calcium channel blockers and medications that influence the activity of different neurotransmitter systems (e.g., dopamine, serotonin, and GABA), influence tDCS effects [123]. In the case of dopamine, several studies have been conducted [124–129]. Although still not very clear, it seems that the effects depend on the dose applied and the targeted receptor. Overall, the results suggest that DRD1 play a role both in the tDCS after effects produced for both the cathode and the anode electrodes [123]. It has also been demonstrated that tDCS can reduce the consumption of analgesic medication, including opioid [130]. Therefore, it can be a therapeutic alternative to the use of opioids, promoting some degree of analgesia, without significant side effects (e.g., tolerance and dependence related to opioids). Moreover, the possible ability of tDCS to activate other modulatory systems would be important in the treatment of chronic pain syndromes. Nonetheless, those results must be further expanded and confirmed in clinical studies in order to establish the specific contribution of each of those systems to neuromodulation-induced analgesia [119].

## 5. Conclusions

Despite the vast recent literature demonstrating the analgesic effects of neuromodulatory techniques, the neuromechanisms involved in both tDCS and TMS-induced analgesia remain largely uncovered. In this regard, a possible role of endogenous modulatory mechanisms (e.g., opioidergic and dopaminergic) has emerged in the last few years. Interestingly, altered functioning of the same systems has been reported in different chronic pain syndromes, suggesting that noninvasive neuromodulatory methods could act, at least in part, by reverting neuroplastic changes related to chronic pain. Nonetheless, future studies will be necessary to clarify the specific impact of each modulatory system and the precise mechanisms of the analgesic effects provided by different techniques of noninvasive neuromodulation.

## Figures and Tables

**Table 1:** A summary of the main findings of the studies investigating the effects of tDCS and TMS on the opioidergic system.

Opioidergic system				
Reference	Design	Population (*n*)	Intervention	Result
Gabis et al., 2003	Randomized double-blind placebo-controlled study	Chronic back pain patients *N*=20	Active or placebo. Transcranial electrical stimulation (TCES).	Increased levels of beta-endorphin in seven out of the ten patients from the treatment group.

De Andrade et al., 2011	Crossover randomized double-blind placebo-controlled study	Healthy volunteers *N*=36	Two groups of active TMS (right M1 or DLPFC/PMC) and one group of sham TMS (M1 or DLPFC/PMC), after a pretreatment with intravenous saline or naloxone.	Naloxone injection significantly reduced the analgesic effects of M1-TMS. However, it did not affect the effects of DLPFC-rTMS or sham rTMS.

Taylor et al., 2012	Crossover randomized double-blind placebo-controlled study	Healthy volunteers *N*=14	Active or sham left DLPFC-TMS, after a pretreatment with intravenous saline or naloxone.	Naloxone pretreatment significantly decreased the analgesic effects of active TMS.

DosSantos et al., 2014	Observational study	Healthy volunteers *N*=9	Study investigating the effects of M1-tDCS on the mu-opioid system through PET.	Placebo tDCS induced a reduction in the availability of MOR in the thalamus, precuneus, and PAG. Active tDCS induced MOR activation in the PAG and precuneus and left prefrontal cortex.

Lamusuo et al., 2017	Crossover randomized double-blind placebo-controlled study	Healthy volunteers *N*=10	Active or sham rTMS applied to the right M1/S1 cortex, combined with opioidergic/dopaminergic evaluation though PET.	Lower opioid receptor availability associated with active rTMS, when compared to sham, in the right ventral striatum, PFC, medial orbitofrontal cortex, ACC, DLPFC, insula, and precentral and superior temporal gyrus. No changes in striatal dopamine D2 receptor.

**Table 2:** A summary of the main findings of the studies investigating the effects of TMS on the dopaminergic system.

Dopaminergic system				
Study	Design	Population (*n*)	Intervention	Result
Fonteneau et al., 2018	Double blind sham-controlled study	Healthy volunteers. *N*=32 Sham group = 18 Active group = 14	Single tDCS (tDCS) session applied over the dorsolateral prefrontal cortex Dynamic PET scan using [11C] raclopride binding	Active tDCS induced a significant decrease in [11C] raclopride BPND in the striatum when compared with sham tDCS

Jääskeläinen et al., 2014	Clinical study	Healthy volunteers (*N* = 29) Patients with neuropathic orofacial pain (*N*=16) Subjects were genotyped for the DRD2 gene 957C > *T* and catechol-O-methyl transferase (COMT) protein Val158Met polymorphisms	Navigated rTMS applied to the S1/M1 cortex. Evaluation of thermal sensitivity and analgesic efficacy.	In healthy subjects, both innocuous and noxious thermal detection thresholds were lowest in 957TT homozygotes rTMS showed analgesic effect only in 957TT homozygote genotype. In patients, the prevalence of 957TT homozygote genotype was higher than in the healthy population. These reported more severe pain than patients with other genotypes

**Table 3:** A summary of the main findings of the studies investigating the effects of TMS on the glutamatergic system.

Glutamatergic system				
Study	Design	Population (*n*)	Intervention	Result
Fregni et al., 2011	Crossover randomized double-blind placebo-controlled study	Chronic pancreatitis/visceral pain *N*=17 Sham group: 8 Real group: 9	Ten sessions of real or sham rTMS of SII spectroscopy evaluation.	No significant changes in glutamate and N-acetyl aspartate (NAA) levels for either left or right SII-rTMS in the sham group Significant increases in glutamate and NAA levels in the active group

De Andrade et al., 2013	Crossover randomized double-blind placebo-controlled study	Healthy volunteers. *N*=36	Active rTMS of the right M1; active rTMS of the right DLPFC/PMC; or sham, after either intravenous saline or ketamine pretreatment	Ketamine significantly decreased the analgesic effects of both M1- and DLPFC/PMC-TMS

Wischnewski et al., 2018	Clinical study	Healthy volunteers. *N*=11	20 Hz beta tACS to M1, after intake of dextromethorphan (DMO) or placebo.	Motor evoked potential significantly increased after tACS in placebo group compared with baseline. However, this effect was not found in the DMO group. Resting-state beta oscillatory activity increases when compared to baseline in the placebo group, but not in the DMO group

**Table 4:** A summary of the main findings of the studies investigating the effects of TMS on the serotonergic system.

Serotonergic system				
Study	Design	Population (*n*)	Intervention	Result
Kuo et al., 2016	Crossover randomized double-blind placebo-controlled study	Healthy volunteers. *N*=12	Four sessions of active tDCS of M1, after intake of placebo, dextromethorphan or citalopram	Chronic administration of citalopram prolonged and enhanced the LTP driven by anodal stimulation. Furthermore, it converted the LTD related to cathodal stimulation into facilitation. Both effects were reverted by dextromethorphan administration.

## References

[1] Merskey H., Bogduk N. (1994). *International Association for the Study of P. Classification of Chronic Pain: Descriptions of Chronic Pain Syndromes and Definitions of Pain Terms*.

[2] Sternbach R. A. (1981). Chronic pain as a disease entity. *Triangle*.

[3] Gaskin D. J., Richard P. (2012). The economic costs of pain in the United States. *Journal of Pain*.

[4] Peyron R., Laurent B., García-Larrea L. (2000). Functional imaging of brain responses to pain. A review and meta-analysis (2000). *Neurophysiologie Clinique*.

[5] Mouraux A., Diukova A., Lee M. C., Wise R. G., Iannetti G. D. (2011). A multisensory investigation of the functional significance of the “pain matrix”. *NeuroImage*.

[6] Mao J. (2012). Current challenges in translational pain research. *Trends in Pharmacological Sciences*.

[7] Vaseghi B., Zoghi M., Jaberzadeh S. (2014). Does anodal transcranial direct current stimulation modulate sensory perception and pain? A meta-analysis study. *Clinical Neurophysiology*.

[8] Marlow N. M., Bonilha H. S., Short E. B. (2013). Efficacy of transcranial direct current stimulation and repetitive transcranial magnetic stimulation for treating fibromyalgia syndrome: a systematic review. *Pain Practice*.

[9] Chen C. C., Chuang Y. F., Huang A. C., Chen C. K., Chang Y. J. (2016). The antalgic effects of non-invasive physical modalities on central post-stroke pain: a systematic review. *Journal of Physical Therapy Science*.

[10] Brunoni A. R., Amadera J., Berbel B., Volz M. S., Rizzerio B. G., Fregni F. (2011). A systematic review on reporting and assessment of adverse effects associated with transcranial direct current stimulation. *International Journal of Neuropsychopharmacology*.

[11] Valle A., Roizenblatt S., Botte S. (2009). Efficacy of anodal transcranial direct current stimulation (tDCS) for the treatment of fibromyalgia: results of a randomized, sham-controlled longitudinal clinical trial. *Journal of Pain Management*.

[12] Fregni F., Boggio P., Nitsche M. (2005). Anodal transcranial direct current stimulation of prefrontal cortex enhances working memory. *Experimental Brain Research*.

[13] Boggio P., Zaghi S., Fregni F. (2009). Modulation of emotions associated with images of human pain using anodal transcranial direct current stimulation (tDCS). *Neuropsychologia*.

[14] Kolbinger H. M., Höflich G., Hufnagel A., Müller H.-J., Kasper S. (1995). Transcranial magnetic stimulation (TMS) in the treatment of major depression—a pilot study. *Human Psychopharmacology: Clinical and Experimental*.

[15] Webster B. R., Celnik P. A., Cohen L. G. (2006). Noninvasive brain stimulation in stroke rehabilitation. *NeuroRx*.

[16] Kellaway P. (1946). The part played by electric fish in the early history of bioelectricity and electrotherapy. *Bulletin of the History of Medicine*.

[17] Bindman L. J., Lippold O. C., Redfearn J. W. (1964). The action of brief polarizing currents on the cerebral cortex of the rat (1) during current flow and (2) in the production of long-lasting after-effects. *Journal of Physiology*.

[18] Lippold O. C., Redfearn J. W. (1964). Mental changes resulting from the passage of small direct currents through the human brain. *British Journal of Psychiatry*.

[19] Redfearn J. W., Lippold O. C., Costain R. (1964). A preliminary account of the clinical effects of polarizing the brain in certain psychiatric disorders. *British Journal of Psychiatry*.

[20] Sheffield L. J., Mowbray R. M. (1968). The effects of polarization on normal subjects. *British Journal of Psychiatry*.

[21] Parent A. (2004). Giovanni Aldini: from animal electricity to human brain stimulation. *Canadian Journal of Neurological Sciences*.

[22] Barker A. T., Jalinous R., Freeston I. L. (1985). Non-invasive magnetic stimulation of human motor cortex. *The Lancet*.

[23] Priori A., Berardelli A., Rona S., Accornero N., Manfredi M. (1998). Polarization of the human motor cortex through the scalp. *NeuroReport*.

[24] Nitsche M., Paulus W. (2000). Excitability changes induced in the human motor cortex by weak transcranial direct current stimulation. *Journal of Physiology*.

[25] O’Connell N. E., Marston  L., Spencer S., DeSouza L. H., Wand B. M. (2018). Non–invasive brain stimulation techniques for chronic pain. *Cochrane Database of Systematic Reviews*.

[26] de Andrade D. C., Mhalla A., Adam F., Texeira M. J., Bouhassira D. (2011). Neuropharmacological basis of rTMS-induced analgesia: the role of endogenous opioids. *Pain*.

[27] DosSantos M. F., Love T. M., Martikainen I. K. (2012). Immediate effects of tDCS on the μ-opioid system of a chronic pain patient. *Frontiers in Psychiatry*.

[28] DosSantos M. F., Martikainen I. K., Nascimento T. D. (2014). Building up analgesia in humans via the endogenous μopioid system by combining placebo and active tDCS: a preliminary report. *PLoS One*.

[29] Taylor J. J., Borckardt J. J., Canterberry M. (2013). Naloxone-reversible modulation of pain circuitry by left prefrontal rTMS. *Neuropsychopharmacology*.

[30] Lamusuo S., Hirvonen J., Lindholm P. (2017). Neurotransmitters behind pain relief with transcranial magnetic stimulation - positron emission tomography evidence for release of endogenous opioids. *European Journal of Pain*.

[31] Ko J. H., Monchi O., Ptito A., Bloomfield P., Houle S., Strafella A. P. (2008). Theta burst stimulation-induced inhibition of dorsolateral prefrontal cortex reveals hemispheric asymmetry in striatal dopamine release during a set-shifting task: a TMS-[(11)C]raclopride PET study. *European Journal of Neuroscience*.

[32] Pereira E. A., Wang S., Peachey T. (2013). Elevated gamma band power in humans receiving naloxone suggests dorsal periaqueductal and periventricular gray deep brain stimulation produced analgesia is opioid mediated. *Experimental Neurology*.

[33] Melzack R. (1999). Pain—an overview. *Acta Anaesthesiologica Scandinavica*.

[34] Kulkarni B., Bentley D. E., Elliott R. (2005). Attention to pain localization and unpleasantness discriminates the functions of the medial and lateral pain systems. *European Journal of Neuroscience*.

[35] Apkarian A. V., Bushnell M. C., Treede R. D., Zubieta J. K. (2005). Human brain mechanisms of pain perception and regulation in health and disease. *European Journal of Pain*.

[36] Morton D. L., Sandhu J. S., Jones A. K. (2016). Brain imaging of pain: state of the art. *Journal of Pain Research*.

[37] Legrain V., Iannetti G. D., Plaghki L., Mouraux A. (2011). The pain matrix reloaded: a salience detection system for the body. *Progress in Neurobiology*.

[38] Iannetti G., Mouraux A. (2010). From the neuromatrix to the pain matrix (and back). *Experimental Brain Research*.

[39] Mouraux A., Iannetti G. D. (2009). Nociceptive laser-evoked brain potentials do not reflect nociceptive-specific neural activity. *Journal of Neurophysiology*.

[40] Staud R. (2012). Abnormal endogenous pain modulation is a shared characteristic of many chronic pain conditions. *Expert Review of Neurotherapeutics*.

[41] Melzack R., Wall P. D. (1965). Pain mechanisms: a new theory. *Science*.

[42] Sherrington C. S., Sowton S. C. (1915). Observations on reflex responses to single break-shocks. *Journal of Physiology*.

[43] Sillery E., Bittar R. G., Robson M. D. (2005). Connectivity of the human periventricular-periaqueductal gray region. *Journal of Neurosurgery*.

[44] Hosobuchi Y., Adams J. E., Linchitz R. (1977). Pain relief by electrical stimulation of the central gray matter in humans and its reversal by naloxone. *Science*.

[45] Mantyh P. W. (1983). The spinothalamic tract in the primate: a re-examination using wheatgerm agglutinin conjugated to horseradish peroxidase. *Neuroscience*.

[46] Fields H. (2004). State-dependent opioid control of pain. *Nature Reviews Neuroscience*.

[47] Swett J. E., McMahon S. B., Wall P. D. (1985). Long ascending projections to the midbrain from cells of lamina I and nucleus of the dorsolateral funiculus of the rat spinal cord. *Journal of Comparative Neurology*.

[48] Tracey I., Mantyh P. W. (2007). The cerebral signature for pain perception and its modulation. *Neuron*.

[49] Valet M., Sprenger T., Boecker H. (2004). Distraction modulates connectivity of the cingulo-frontal cortex and the midbrain during pain--an fMRI analysis. *Pain*.

[50] Heinricher M. M., Tavares I., Leith J. L., Lumb B. M. (2009). Descending control of nociception: specificity, recruitment and plasticity. *Brain Research Reviews*.

[51] Wagner T., Fregni F., Fecteau S., Grodzinsky A., Zahn M., Pascual-Leone A. (2007). Transcranial direct current stimulation: a computer-based human model study. *NeuroImage*.

[52] Eippert F., Bingel U., Schoell E. D. (2009). Activation of the opioidergic descending pain control system underlies placebo analgesia. *Neuron*.

[53] Benedetti F., Mayberg H. S., Wager T. D., Stohler C. S., Zubieta J. K. (2005). Neurobiological mechanisms of the placebo effect. *Journal of Neuroscience*.

[54] Jones A. K. P., Brown C. A. (2018). Predictive mechanisms linking brain opioids to chronic pain vulnerability and resilience. *British Journal of Pharmacology*.

[55] Goffaux P., Redmond W. J., Rainville P., Marchand S. (2007). Descending analgesia--when the spine echoes what the brain expects. *Pain*.

[56] Pecina M., Zubieta J. K. (2018). Expectancy modulation of opioid neurotransmission. *International Review of Neurobiology*.

[57] Bingel U., Lorenz J., Schoell E., Weiller C., Büchel C. (2006). Mechanisms of placebo analgesia: rACC recruitment of a subcortical antinociceptive network. *Pain*.

[58] Navratilova E., Atcherley C. W., Porreca F. (2015). Brain circuits encoding reward from pain relief. *Trends in Neurosciences*.

[59] Baliki M. N., Petre B., Torbey S. (2012). Corticostriatal functional connectivity predicts transition to chronic back pain. *Nature Neuroscience*.

[60] Harris R. E., Clauw D. J., Scott D. J., McLean S. A., Gracely R. H., Zubieta J. K. (2007). Decreased central mu-opioid receptor availability in fibromyalgia. *Journal of Neuroscience*.

[61] Melzack R. (1987). The short-form McGill pain questionnaire. *Pain*.

[62] Willoch F., Schindler F., Wester H. J. (2004). Central poststroke pain and reduced opioid receptor binding within pain processing circuitries: a [11C]diprenorphine PET study. *Pain*.

[63] Jones A. K., Cunningham V. J., Ha-Kawa S. (1994). Changes in central opioid receptor binding in relation to inflammation and pain in patients with rheumatoid arthritis. *British Journal of Rheumatology*.

[64] Brown C. A., Matthews J., Fairclough M. (2015). Striatal opioid receptor availability is related to acute and chronic pain perception in arthritis: does opioid adaptation increase resilience to chronic pain?. *Pain*.

[65] Wey H. Y., Catana C., Hooker J. M. (2014). Simultaneous fMRI-PET of the opioidergic pain system in human brain. *NeuroImage*.

[66] Schrepf A., Harper D. E., Harte S. E. (2016). Endogenous opioidergic dysregulation of pain in fibromyalgia: a PET and fMRI study. *Pain*.

[67] Navratilova E., Porreca F. (2014). Reward and motivation in pain and pain relief. *Nature Neuroscience*.

[68] DaSilva A. F., Nascimento T. D., DosSantos M. F. (2014). Association of *μ*-opioid activation in the prefrontal cortex with spontaneous migraine attacks - brief report I. *Annals of Clinical and Translational Neurology*.

[69] Afridi S. K., Matharu M. S., Lee L. (2005). A PET study exploring the laterality of brainstem activation in migraine using glyceryl trinitrate. *Brain*.

[70] Denuelle M., Fabre N., Payoux P., Chollet F., Geraud G. (2007). Hypothalamic activation in spontaneous migraine attacks. *Headache*.

[71] Nascimento T. D., DosSantos M. F., Lucas S. (2014). *μ*-Opioid activation in the midbrain during migraine allodynia—brief report II. *Ann Clin Transl Neurol*.

[72] Poras H., Bonnard E., Dangé E., Fournié-Zaluski M. C., Roques B. P. (2014). New orally active dual enkephalinase inhibitors (DENKIs) for central and peripheral pain treatment. *Journal of Medicinal Chemistry*.

[73] Thompson S. J., Pitcher M. H., Stone L. S. (2018). Chronic neuropathic pain reduces opioid receptor availability with associated anhedonia in rat. *Pain*.

[74] Loggia M. L. (2018). Chronic pain and opioid receptor availability: disentangling the molecular contributions and the “chicken or the egg” dilemma. *Pain*.

[75] Wood P. B., Patterson J. C., Sunderland J. J., Tainter K. H., Glabus M. F., Lilien D. L. (2007). Reduced presynaptic dopamine activity in fibromyalgia syndrome demonstrated with positron emission tomography: a pilot study. *Journal of Pain*.

[76] Leknes S., Tracey I. (2008). A common neurobiology for pain and pleasure. *Nature Reviews Neuroscience*.

[77] Missale C., Nash S. R., Robinson S. W., Jaber M., Caron M. G. (1998). Dopamine receptors: from structure to function. *Physiological Reviews*.

[78] Scott D. J., Heitzeg M. M., Koeppe R. A., Stohler C. S., Zubieta J. K. (2006). Variations in the human pain stress experience mediated by ventral and dorsal basal ganglia dopamine activity. *Journal of Neuroscience*.

[79] Ballantyne J. C., Sullivan M. D. (2017). Discovery of endogenous opioid systems: what it has meant for the clinician’s understanding of pain and its treatment. *Pain*.

[80] DaSilva A. F., Nascimento T. D., Jassar H. (2017). Dopamine D2/D3 imbalance during migraine attack and allodynia in vivo. *Neurology*.

[81] Martikainen I. K., Nuechterlein E. B., Peciña M. (2015). Chronic back pain is associated with alterations in dopamine neurotransmission in the ventral striatum. *Journal of Neuroscience*.

[82] Wood P. B., Schweinhardt P., Jaeger E. (2007). Fibromyalgia patients show an abnormal dopamine response to pain. *European Journal of Neuroscience*.

[83] Hagelberg N., Forssell H., Aalto S. (2003). Altered dopamine D2 receptor binding in atypical facial pain. *Pain*.

[84] Hagelberg N., Forssell H., Rinne J. O. (2003). Striatal dopamine D1 and D2 receptors in burning mouth syndrome. *Pain*.

[85] Dawson A., Stensson N., Ghafouri B. (2016). Dopamine in plasma—a biomarker for myofascial TMD pain?. *Journal of Headache and Pain*.

[86] Koob G. F., Volkow N. D. (2010). Neurocircuitry of addiction. *Neuropsychopharmacology*.

[87] Drake C. T., Milner T. A. (1999). Mu opioid receptors are in somatodendritic and axonal compartments of GABAergic neurons in rat hippocampal formation. *Brain Research*.

[88] Reisi Z., Haghparast A., Pahlevani P., Shamsizadeh A. (2014). Interaction between the dopaminergic and opioidergic systems in dorsal hippocampus in modulation of formalin-induced orofacial pain in rats. *Pharmacology Biochemistry and Behavior*.

[89] Young N. A., Sharma M., Deogaonkar M. (2014). Transcranial magnetic stimulation for chronic pain. *Neurosurgery Clinics of North America*.

[90] Chervyakov A. V., Chernyavsky A. Y., Sinitsyn D. O., Piradov M. A. (2015). Possible mechanisms underlying the therapeutic effects of transcranial magnetic stimulation. *Frontiers in Human Neuroscience*.

[91] Esser S. K., Huber R., Massimini M., Peterson M. J., Ferrarelli F., Tononi G. (2006). A direct demonstration of cortical LTP in humans: a combined TMS/EEG study. *Brain Research Bulletin*.

[92] Chen R., Classen J., Gerloff C. (1997). Depression of motor cortex excitability by low-frequency transcranial magnetic stimulation. *Neurology*.

[93] DaSilva A. F., Truong D. Q., DosSantos M. F., Toback R. L., Datta A., Bikson M. (2015). State-of-art neuroanatomical target analysis of high-definition and conventional tDCS montages used for migraine and pain control. *Frontiers in Neuroanatomy*.

[94] DosSantos M. F., Ferreira N., Toback R. L., Carvalho A. C., DaSilva A. F. (2016). Potential mechanisms supporting the value of motor cortex stimulation to treat chronic pain syndromes. *Frontiers in Neuroscience*.

[95] Tzabazis A., Aparici C. M., Rowbotham M. C., Schneider M. B., Etkin A., Yeomans D. C. (2013). Shaped magnetic field pulses by multi-coil repetitive transcranial magnetic stimulation (rTMS) differentially modulate anterior cingulate cortex responses and pain in volunteers and fibromyalgia patients. *Molecular Pain*.

[96] Sacco P., Prior M., Poole H., Nurmikko T. (2014). Repetitive transcranial magnetic stimulation over primary motor vs non-motor cortical targets; effects on experimental hyperalgesia in healthy subjects. *BMC Neurology*.

[97] Taylor J. J., Borckardt J. J., George M. S. (2012). Endogenous opioids mediate left dorsolateral prefrontal cortex rTMS-induced analgesia. *Pain*.

[98] Lefaucheur J. P., André-Obadia N., Antal A. (2014). Evidence-based guidelines on the therapeutic use of repetitive transcranial magnetic stimulation (rTMS). *Clinical Neurophysiology*.

[99] Moisset X., de Andrade D. C., Bouhassira D. (2015). From pulses to pain relief: an update on the mechanisms of rTMS-induced analgesic effects. *European Journal of Pain*.

[100] Maarrawi J., Peyron R., Mertens P. (2007). Motor cortex stimulation for pain control induces changes in the endogenous opioid system. *Neurology*.

[101] Medeiros L. F., de Souza I. C., Vidor L. P. (2012). Neurobiological effects of transcranial direct current stimulation: a review. *Frontiers in Psychiatry*.

[102] Nitsche M. A., Cohen L. G., Wassermann E. M. (2008). Transcranial direct current stimulation: state of the art 2008. *Brain Stimulation*.

[103] Besson J. M. (1999). The neurobiology of pain. *The Lancet*.

[104] Feng Y., He X., Yang Y., Chao D., Lazarus L. H., Xia Y. (2012). Current research on opioid receptor function. *Current Drug Targets*.

[105] Alexandre F. M., DaSilva M. F. D. (2016). *Transcranial Direct Current Stimulation in Neuropsychiatric Disorders*.

[106] Skolnick M. H., Wilson O. B., Hamilton R. F. (1989). Low current electrostimulation produces naloxone-reversible analgesia in rats. *Stereotactic and Functional Neurosurgery*.

[107] Gabis L., Shklar B., Geva D. (2003). Immediate influence of transcranial electrostimulation on pain and beta-endorphin blood levels: an active placebo-controlled study. *American Journal of Physical Medicine and Rehabilitation*.

[108] Maarrawi J., Peyron R., Mertens P. (2007). Differential brain opioid receptor availability in central and peripheral neuropathic pain. *Pain*.

[109] Strafella A. P., Paus T., Barrett J., Dagher A. (2001). Repetitive transcranial magnetic stimulation of the human prefrontal cortex induces dopamine release in the caudate nucleus. *Journal of Neuroscience*.

[110] Strafella A. P., Paus T., Fraraccio M., Dagher A. (2003). Striatal dopamine release induced by repetitive transcranial magnetic stimulation of the human motor cortex. *Brain*.

[111] Cho S. S., Strafella A. P. (2009). rTMS of the left dorsolateral prefrontal cortex modulates dopamine release in the ipsilateral anterior cingulate cortex and orbitofrontal cortex. *PLoS One*.

[112] Evinger C., Basso M. A., Manning K. A., Sibony P. A., Pellegrini J. J., Horn A. K. (1993). A role for the basal ganglia in nicotinic modulation of the blink reflex. *Experimental Brain Research*.

[113] de la Fuente-Fernández R. (2009). The placebo-reward hypothesis: dopamine and the placebo effect. *Parkinsonism and Related Disorders*.

[114] Jääskeläinen S. K., Lindholm P., Valmunen T. (2014). Variation in the dopamine D2 receptor gene plays a key role in human pain and its modulation by transcranial magnetic stimulation. *Pain*.

[115] Hirvonen M., Laakso A., Någren K., Rinne J. O., Pohjalainen T., Hietala J. (2004). C957T polymorphism of the dopamine D2 receptor (DRD2) gene affects striatal DRD2 availability in vivo. *Molecular Psychiatry*.

[116] Hirvonen M. M., Laakso A., Någren K., Rinne J. O., Pohjalainen T., Hietala J. (2009). C957T polymorphism of dopamine D2 receptor gene affects striatal DRD2 in vivo availability by changing the receptor affinity. *Synapse*.

[117] Hirvonen M. M., Lumme V., Hirvonen J. (2009). C957T polymorphism of the human dopamine D2 receptor gene predicts extrastriatal dopamine receptor availability in vivo. *Progress in Neuro-Psychopharmacology and Biological Psychiatry*.

[118] Ciampi de Andrade D., Mhalla A., Adam F., Texeira M. J., Bouhassira D. (2014). Repetitive transcranial magnetic stimulation induced analgesia depends on N-methyl-D-aspartate glutamate receptors. *Pain*.

[119] Souza A., Martins D. F., Medeiros L. F. (2018). Neurobiological mechanisms of antiallodynic effect of transcranial direct current stimulation (tDCS) in a mice model of neuropathic pain. *Brain Research*.

[120] Zhang J., Hoffert C., Vu H. K., Groblewski T., Ahmad S., O’Donnell D. (2003). Induction of CB2 receptor expression in the rat spinal cord of neuropathic but not inflammatory chronic pain models. *European Journal of Neuroscience*.

[121] Slivicki R. A., Xu Z., Kulkarni P. M. (2017). Positive allosteric modulation of cannabinoid receptor type 1 suppresses pathological pain without producing tolerance or dependence. *Biological Psychiatry*.

[122] Hughes S. W., Ali M., Sharma P., Insan N., Strutton P. H. (2018). Frequency-dependent top-down modulation of temporal summation by anodal transcranial direct-current stimulation of the primary motor cortex in healthy adults. *European Journal of Pain*.

[123] McLaren M. E., Nissim N. R., Woods A. J. (2018). The effects of medication use in transcranial direct current stimulation: a brief review. *Brain Stimulation*.

[124] Kuo M. F., Paulus W., Nitsche M. A. (2008). Boosting focally-induced brain plasticity by dopamine. *Cerebral Cortex*.

[125] Monte-Silva K., Liebetanz D., Grundey J., Paulus W., Nitsche M. A. (2010). Dosage-dependent non-linear effect of L-dopa on human motor cortex plasticity. *Journal of Physiology*.

[126] Nitsche M. A., Jaussi W., Liebetanz D., Lang N., Tergau F., Paulus W. (2004). Consolidation of human motor cortical neuroplasticity by D-cycloserine. *Neuropsychopharmacology*.

[127] Nitsche M. A., Lampe C., Antal A. (2006). Dopaminergic modulation of long-lasting direct current-induced cortical excitability changes in the human motor cortex. *European Journal of Neuroscience*.

[128] Monte-Silva K., Kuo M. F., Thirugnanasambandam N., Liebetanz D., Paulus W., Nitsche M. A. (2009). Dose-dependent inverted U-shaped effect of dopamine (D2-like) receptor activation on focal and nonfocal plasticity in humans. *Journal of Neuroscience*.

[129] Nitsche M. A., Kuo M. F., Grosch J., Bergner C., Monte-Silva K., Paulus W. (2009). D1-receptor impact on neuroplasticity in humans. *Journal of Neuroscience*.

[130] Khedr E. M., Sharkawy E. S. A., Attia A. M. A., Ibrahim Osman N. M., Sayed Z. M. (2017). Role of transcranial direct current stimulation on reduction of postsurgical opioid consumption and pain in total knee arthroplasty: double randomized clinical trial. *European Journal of Pain*.

